# Factors influencing the addiction characteristics of non-suicidal self-injurious behaviors in adolescents: A case-control study

**DOI:** 10.3389/fpsyt.2022.1033242

**Published:** 2022-12-01

**Authors:** Junhong Zhu, Rui Qian, Hao Zhong, Yi Li, Xuebing Liu, Jun Ma

**Affiliations:** ^1^Department of Psychiatry, Wuhan Mental Health Center, Wuhan, China; ^2^Wuhan Hospital for Psychotherapy, Wuhan, China

**Keywords:** adolescent, NSSI, behavioral addiction, parental rearing style, social media dependency, video game addiction

## Abstract

**Background:**

Many studies have shown that in the context of public health emergencies, the incidence rate of adolescent non-suicidal self-injury (NSSI) patients increased dramatically. This paper aims to characterize the behavioral characteristics of adolescent NSSI and analyze the influencing factors of NSSI behavior addiction characteristics.

**Methods:**

Our research was a case-control study which included 84 adolescents with NSSI (female *vs*. male: 59 *vs*. 25) and 84 healthy controls (female *vs*. male: 53 *vs*. 31). All the participants enrolled were aged 12-18 years. The differences in the scores of the following five scales were compared between the case and control groups: Egna Minnen Barndoms Uppfostran (EMBU), Perceived Social Support Scale (PSSS), Perceived Stress Scale (PSS); Bergen Social Media Addiction Scale (BSMAS) and Video Game Dependence Scale (VDG-S). The characteristics of NSSI behavior of the study group were evaluated using the Ottawa Self Inventory Chinese Revised Edition (OSIC). And a binary logistic regression model was developed to analyze the factors that influence adolescent NSSI behavioral addiction characteristics.

**Results:**

In the study group, the emotional warmth scores in the father’s and mother’s rearing style scores in the EMBU were significantly lower than the controls. The BSMAS and VDG-S scores were significantly higher than those in the control group. 38 cases of NSSI with addiction characteristics accounted for 45.24% in the study group. The risk factors for NSSI addiction traits were as follows: female, single-child, high level of VDG-S scores, high scores of excessive interferences in father’s rearing style, and high scores of punishments and excessive interferences in mother’s rearing style score.

**Conclusion:**

Female, only child, internet addiction, and negative parenting styles were predictors of NSSI behavioral addiction characteristics in adolescents. Targeted coping strategies should be developed to reduce the occurrence and development of self-injurious behavior, especially for female adolescents with Internet dependence in one-child families with negative parenting styles.

## Introduction

The term non-suicidal self-injury (NSSI) refers to behaviors that do not aim to end a person’s life but intentionally cause body tissue damage by themselves and are not recognized by society (1), which is especially common in adolescence (2). NSSI is described as having an addiction characteristics or functions (3, 4), which means the behavior is out of control and recurring. As with substance dependence, NSSI causes abnormalities in a wide range of amygdala circuits (5). The reported prevalence of NSSI in adolescents ranges from 11.5 to 47.1% (6–8), and continues showing a gradual increase (6). Although the ultimate goal is not to end life, the abnormal mortality of adolescent NSSI patients in the future is about 9 times higher than that of the general population, the risk of suicide increases by 17.5 times, and acute alcohol and drug poisoning increases by 34 times (9). Despite the seriousness of the risk, the etiology of NSSI remains unknown and the factors associated with the etiology are complex, which make it difficult to provide targeted interventions.

The global pandemic of Coronavirus disease 2019 (COVID-19) has had a broad and far-reaching impact on the general population’s mental health (10). Adolescents are more vulnerable to the negative impact of the epidemic spread than adults because of their immature development of psychological defense mechanisms and lacking the experience of dealing with major public health and safety emergencies. A report confirmed 23 emergency services for children and adolescents in 10 countries between March and April 2020, which found that the number of children and adolescents seeking emergency psychiatric services due to self-harm increased by 33% compared with the same period in 2019 (10). Before the epidemic, the overall prevalence of non-suicidal self-injury (NSSI) among Chinese middle school students was 22.37% (11). In contrast, during the COVID-19 outbreak (February 28 to March 11, 2020), data from the Taiwan Province of China found that the prevalence of NSSI among junior high school students was 40.9% (12). Simultaneously, the proportion of NSSI among hospitalized adolescents with mental disorders in China has increased from 29.2% in 2016 to 92.5% in 2020 and 95.9% in 2021 (13).

Another important phenomenon that accompanied the epidemic secondary to the epidemic was the more frequent interactions between the general population and the Internet. During the COVID-19 outbreak, the overall prevalence of Internet addiction in the general population was 36.7% (14), with nearly 50% of subjects reporting increased dependence on Internet use (15). In China, adolescents need to be taught online during the epidemic. They are exposed to the Internet longer than usual, and as a result, the proportion of Internet addiction and gaming behavior among them has changed (16). A 2019 study showed that the prevalence of Internet addiction among Chinese adolescents has reached 15.3% (17), while the prevalence among junior high school students after the epidemic was 24.4–31.2% (18, 19).

Negative parenting styles and adverse childhood experiences have been reported to contribute to the emergence of NSSI behaviors in adolescents. For example, a large cross-sectional study from Yunnan Province, China, with school-based secondary school students, reported that negative parenting styles were associated with adolescent NSSI behavior (7). Inadequate social support is another crucial correlate of NSSI (20). In contrast, a good social support system can also be an essential measure in improving the mental health of adolescent students (21). In addition to the above reasons, another relatively well-reported one is the negative impact of excessive Internet use on adolescent NSSI behavior (22, 23). Offline social support is reported to show a negative association with NSSI (24). However, in Chian, during the outbreak and at a time when the epidemic is being managed on a regular basis, online instruction is the only or primary way for adolescents to maintain their education, which increases the risk of their overuse of the Internet and increases the risk that Internet addiction and NSSI behaviors are intertwined and affect each other.

It is regretful that most of the current research on adolescent NSSI behaviors is based on epidemiological surveys, and relatively few studies have been conducted on adolescent patients hospitalized as a result of NSSI behaviors. In the context of the rapidly increasing prevalence of NSSI in adolescents, the authors hypothesized that the addictive characteristics of inpatient adolescent NSSI behavior are influenced by a variety of factors including parenting style, individual perceptions of stress and social support, and Internet addiction. The purpose of this paper is to explore the factors that influence the behavioral addiction characteristics of hospitalized adolescents with NSSI and to provide some effective and reasonable recommendations for the treatment of NSSI.

## Materials and methods

### Subjects

A total of 84 adolescent patients with NSSI who were admitted to Wuhan Mental Health Center for treatment from February 2021 to April 2022 were included in this study.

#### Inclusion criteria

(1). The Ottawa Self-Injury Inventory Chinese Revised Edition (OSIC) (25) self-injury frequency score: the frequency of self-injury behavior in the past month is not less than 2 points (frequently), and the frequency of self-injury behavior in the past 6 months is not less than 2 points (once a month).
(2). Adolescent patients admitted to the hospital for “self-injury” or accompanied by significant “self-injury behavior.”
(3). Participants are 12–18 years old.
(4). Middle school students who are in school and who are temporarily suspended because of “self-injury behavior.”

#### Exclusion criteria

There are explicit and typical psychotic symptoms such as hallucinations, delusions, and catatonia at the time of admission or a clear history of severe mental diseases such as schizophrenia and bipolar disorder. Patients with intellectual disability, autism, and other reasons for not completing the questionnaire were excluded.

#### Healthy controls

From nearby communities and middle schools, we recruited 84 healthy controls matched with the research group regarding age, gender ratio, education level, the proportion of single-child, and parents’ marital status. We excluded ‘healthy cases’ with self-injury but not seeking medical treatment.

The ethics committee of the Wuhan mental health center approved this study. Informed consent was obtained from the participants themselves and signed by their families or guardians for this study.

### Instruments

The electronic medical record system was used to extract general clinical data from the included patients, such as age, gender, number of siblings, parents’ marital status, education level, household registration type, site of self-injury, and age of first self-injury, which was then recorded item by item in Excel spreadsheet.

Egna Minnen Barndoms Uppfostran (EMBU) is a self-assessment scale that asks subjects to evaluate their parents’ parenting style through recall (26). This scale consists of the father’s and mother’s scales. The father’s scale contains six dimensions: understanding of emotional warmth, severe punishment, excessive interference, preference for subjects, rejection, denial, and overprotection. However, the mother’s scale includes five dimensions compared with the father’s scale, in which overprotection was not included.

The Perceived Social Support Scale (PSSS) (27) is used to measure respondents’ self-reported support from family, friends, and others. This scale has 12 items, and each item is scored on a 7-point scale (1 = strongly disagree; 7 = strongly agree), with higher scores indicating higher levels of perceived support.

The Perceived Stress Scale (PSS) is a self-assessment scale for measuring perceived stress, which has been adapted for use in China by Yang et al. (28). The scale consists of 14 questions reflecting stressful tensions and feelings of loss of control, with items rated on a scale of 0 (never) to 4 (always), with higher scores indicating greater perceived stress.

The Bergen Social Media Addiction Scale (BSMAS) assessed patient dependence on social media. According to the study by Luo et al., we defined a BSMAS score greater than or equal to 24 as social media addiction among Chinese adolescents (29).

The Video Game Dependence Scale (VDG-S) is used to assess patients’ reliance on online games (16). VDG-S primarily assesses subjects’ video game behavior over the past 12 months. The entire scale contains 18 items; each item is scored on a 4-point scale (1 = strongly disagree, 2 = somewhat disagree, 3 = somewhat agree, 4 = strongly agree). The 18 items were further divided into 9 dimensions in each of two items, and at least one item was determined as “strongly agree” by the subjects; the dimension was scored as 1, and the denial was scored as 0. The total score is approximately 5 points to be determined as internet gaming disorder (IGD).

The Ottawa Self-Injury Inventory Chinese Revised Edition (OSIC) is used to assess the specific characteristics of patients’ self-injury behaviors, and this revised version was completed by Chen et al. (13). The text of the revised scale is more concise and efficient and suitable as a clinical and scientific evaluation tool for studying the NSSI behavior of Chinese adolescents. The OSIC can evaluate NSSI thought and behavior frequency, addiction characteristics, and function scale. NSSI thought, and behavior frequency includes three items corresponding to the frequency of NSSI thought and behavior in the past month, the past 6 months, and the past 12 months, respectively. The higher the overall score of the 7 items measuring addiction characteristics, the more addictive the person’s NSSI behavior. Defined three or more of the seven NSSI behaviors with a score of greater than or equal to 2 belonging to the characteristics of addiction. The functional scale consists of three parts: social influence reflects motivations to evoke responses or changes in social contexts, which could enable individuals to attract others’ attention, seek help and gain others’ understanding (items 2, 4, 6, 10, and 14), external emotion regulation is the regulation of emotions through external factors (items 1, 3, 8, 11, and 13) and internal emotion regulation is the regulation of an individual’s own emotions through his or her own internal activities (items 5, 7, 9, 12, and 15). Divided the original total score of the three subscales by the number of items in the subscale to obtain their respective average scores. The scale with the highest average score reflects the most important reason for the individual to engage in non-suicidal self-injury behavior.

We specially designed a web-based questionnaire to collect healthy control data, including demographics, EMBU, PSSS, PSS, BSMAS, and VDG-S.

### Procedures

This study was designed as a case-control study. Except for the type of household, the demographics of the controls and the cases were all matched to each other.

We began evaluating adolescent patients who met the inclusion criteria with relevant questionnaires and scales in February 2021. Data collection was generally completed within 3 days after admission for cooperative patients. For emotionally unstable patients who often cry and cannot cooperate, and patients who cannot be diagnosed, the scale evaluation and data collection would be completed within 14 days at the latest. Patients who were unable to be complete the collection of relevant information within 14 days of admission for any reason were excluded from the study. The average time of data collection for all included patients was 2.96 ± 1.87 days after admission.

While collecting the data of the case group, our self-made online questionnaire was used to collect relevant data on the controls in the community and schools. Participants in the controls were all volunteers. The process for recruiting the controls in the community and nearby schools was as follows: for participants who agreed and were able to sign the informed consent form, they would enter the process of providing relevant information by scanning a pre-made two-dimensional code via their smartphones. Once in the program, participants were first asked to fill in the monthly self-injury frequency and semi-annual self-injury frequency, and if both items were 0, they entered the process of demographic information giving and assessment of target self-reported scales other than OSIC. Otherwise, they were automatically launched from the program.

### Data analysis

The normally distributed continuous measurement data obtained were expressed as the mean and standard deviation, and the categorical variables as counts. The chi-square test was used for comparing rates, the independent samples *t*-test was used for comparing two groups of continuous data, and one-way analysis of variance (ANOVA) was used for comparing the differences among the scores of the three OSIC functional scales. A binary logistic regression model was developed to analyze the influencing factors of NSSI behavioral addiction characteristics. The significance level of all statistical tests was set to *p* < 0.05. Data analysis was performed using the IBM SPSS (version 26.0, SPSS Inc., Chicago, IL, USA), and the figure was plotted using the GraphPad Prism software (version 8.4.3; GraphPad Software Inc., La Jolla, CA, USA).

## Results

### Demographics and general clinical data

At the end point of this study in April 2022, 84 participants were included in the study group that met the inclusion criteria, and 84 healthy controls were recruited from the community and nearby middle school students. For the controls recruitment, a total of 142 participants participated in our questionnaire, of whom 58 were excluded due to incomplete data, and finally, 84 participants were included in the analysis, with a response rate of 59.15%. The demographics and general clinical data of all participants were shown in Table 1.

**TABLE 1:** Participants’ demographics and general clinical data.

Index	Patients (*n* = 84)	Controls (*n* = 84)
Age (years)		
Mean (SD)	15.37 (1.76)	14.89 (3.26)
Range	12-18	12-18
Gender (*n*, %)		
Female	59, 70.2%	53, 63.1%
Male	25, 29.8%	31, 36.9%
Single-child	39, 46.4%	42, 50.0%
Non-single-child	45, 53.6%	42, 50.0%
Educational background (*n*, %)		
Junior high school	35, 41.67%	38, 45.24%
Senior high school	49, 58.33%	46, 54.76%
Type of household (*n*, %)		
Urban status	58, 69%	84, 100%
Rural status	26, 31%	0, 0%
Parents’ marital status (*n*, %)		
Divorced	19, 22.6%	22, 26.19%
Non-divorced	65, 77.4%	74, 73.81%
Age at first self-injury		
Mean (SD)	12.60 (1.47)	
Range	9-15	
Self-injured site (*n*, %)		
Arm part	74, 88.10%	
Arms and Legs	10, 11.90%	
Frequency of NSSI behavior		
Last 1 month	2.43 (0.52)	
Last 6 months	2.42 (0.50)	

### Differences in target scales scores between groups

Compared with the controls, the emotional warmth scores in the father’s and mother’s rearing style scores in the EMBU of the study group were significantly lower (*t = −3.22, p = 0.02*). Meanwhile, the punishment score for the mother’s rearing style was considerably higher than that of the controls (*t = 5.03, p* **<** *0.001*). The score of the study group in support within the family in the PSSS was significantly lower than that of the controls (*t = 5.33, p* **<** *0.001*). Finally, the BSMAS scores and VDG-S scores of the study group were significantly higher than those of the controls (*t = 2.27, p = 0.01*; *t = 2.44, p = 0.02*, respectively) (Table 2).

**TABLE 2:** Differences between the study group and the control group in EMBU, PSSS, PSS, BSMAS, and VDG-S.

	Study group	Control group	*t*	*d*	*p*
EMBU					
Father’s rearing style score					
Emotional warmth	43.71 ± 11.10	45.18 ± 9.98	–3.22	–0.14	0.02*
Punishment	22.85 ± 7.06	21.35 ± 9.20	1.36	0.18	0.11
Excessive interference	21.24 ± 5.01	22.05 ± 7.23	–2.06	–0.13	0.08
Partialism	9.00 ± 3.14	9.02 ± 4.10	–1.00	–0.01	0.29
Rejection	11.38 ± 3.78	10.11 ± 2.96	1.23	0.43	0.54
Overprotection	8.1 ± 2.51	8.31 ± 1.99	–0.98	–0.09	0.39
Mother’s rearing style score					
Emotional warmth	43.04 ± 11.03	48.35 ± 11.43	–2.81	–0.47	0.01*
Punishment	16.37 ± 7.60	13.30 ± 8.83	5.03	0.37	< .001*
Excessive interference	38.97 ± 9.90	39.19 ± 11.21	–2.00	–0.02	0.36
Partialism	16.37 ± 6.80	18.35 ± 6.60	–3.07	–0.30	0.06
Rejection	8.83 ± 3.00	9.12 ± 2.88	–1.01	–0.10	0.40
PSSS					
Total score	49.02 ± 13.19	51.89 ± 10.38	–1.57	–0.24	0.12
Support within the family	16.02 ± 5.67	20.70 ± 5.70	–5.33	–0.82	< .001*
Support outside the family	33.00 ± 9.26	31.19 ± 10.22	1.20	–0.19	0.23
PSS	29.32 ± 5.50	27.15 ± 5.48	2.10	0.40	0.15
BSMAS	16.88 ± 6.05	14.79 ± 5.11	2.27	0.37	0.01*
VDG-S	30.24 ± 14.11	25.87 ± 8.36	2.44	0.38	0.02*

EMBU, egna minnen barndoms uppfostran; PSSS, perceived social support scale; PSS, perceived stress scale; BSMAS, bergen social media addiction scale; VDG-S, video game dependence scale; * *p < 0.05*.

### Differences in the proportion of addiction by group: Based on the Bergen Social Media Addiction Scale scores and the Video Game Dependence Scale scores

Based on the scoring principles of the BSMAS and VDG-S, we calculated the number of individuals eligible for social media addiction and video game addiction and compared the group differences in the prevalence of addiction. Further, we observed no significant difference in prevalence of addiction between the study group and the controls (Table 3).

**TABLE 3:** The prevalence of addictive behaviors between the two groups: Based on BSMAS and VDG-S scores.

	Study group (*n*, %)	Control group (*n*, %)	χ^2^	*p*
BSMAS ≥ 24	14, 16.67%	8, 9.52%	1.88	0.17
VDG-S ≥ 5	6, 7.14%	5, 5.95%	0.10	0.76

BSMAS, bergen social media addiction scale; VDG-S, video game dependence scale.

### Assessment and analysis of Ottawa Self Inventory Chinese Revised Edition and its functional subscales

**FIGURE 1:**
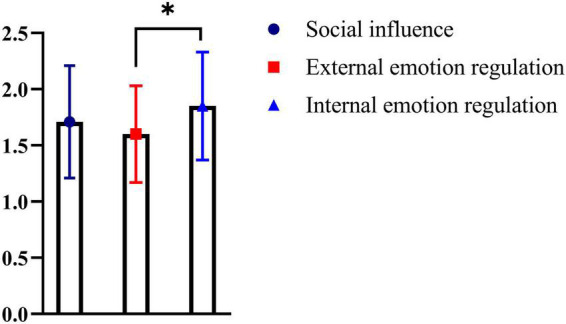
Comparison of differences between scores of three functional scales of OSIC. The average score for internal emotion regulation was significantly higher than external emotion regulation (*p = 0.03*) *p < *0.05.*

According to the scoring rules of the subscale of OSIC addiction characteristics, we found 38 cases of NSSI with addiction characteristics in the study group which accounting for 45.24%. When the average scores of the three OSIC subscales were compared pairwise, it was found that the average score of internal emotion regulation was significantly higher than that of external emotion regulation (*F = 5.84, p = 0.03*) (Figure 1).

### Factors influencing behavioral addiction characteristics of non-suicidal self-injury: Binary logistic regression

Addiction characteristics of NSSI behavior as dependent variable (addiction characteristics = 1, otherwise = 0), gender (0 = female, 1 = male) and age, age at first self-injury, single-child or not (0 = non-single-child, 1 = single-child), type of household (0 = urban status, 1 = rural status), parents’ marital status (0 = divorced, 1 = non-divorced), and the scores of the target scales were used as independent variables (Table 4). Our results found that the risk factors for NSSI addiction characteristics were as follows: female (B = −2.59, *p* = 0.02, OR = 12.50), single-child (B = 2.58, *p* = 0.04, OR = 13.20), VDG-S (B = 0.16, *p* = 0.001, OR = 1.18), excessive interference (B = 0.24, *p* = 0.03, OR = 1.27) for father’s rearing style score, punishment (B = 0.89, *p* = 0.04, OR = 2.43) and excessive interference for mother’s rearing style score (B = 0.12, *p* = 0.02, OR = 1.12).

**TABLE 4:** The factors of addiction characteristics of NSSI behavior: Logistic regression model.

	B (SE)	Wald	OR	95% CI	*p*
Constant	−19.56(7.79)	6.30	0.00		0.01
Gender	−2.59(1.14)	5.11	12.50	0.01-0.71	0.02*
Age	0.12(0.25)	0.21	1.12	0.69-1.84	0.64
Age at first self-injury	−0.22(0.27)	0.67	0.80	0.47-1.36	0.41
Single-child	2.58(1.25)	4.24	13.20	1.13-3.99	0.04*
Type of household	0.94(1.17)	0.66	2.57	0.26-5.23	0.42
Parents’ marital status	2.66(1.32)	4.07	14.34	1.08-9.39	0.24
PSS	0.05(0.10)	0.29	1.05	0.87-1.28	0.59
BSMAS	0.10(0.08)	1.56	1.10	0.95-1.28	0.21
VDG-S	0.16(0.05)	10.56	1.18	1.07-1.30	0.001*
PSSS					
Total score	0.01(0.05)	0.04	1.01	0.91-1.12	0.02
Support within the family	−0.01(0.14)	0.01	0.99	0.75-1.30	0.03*
Support outside the family	0.12(0.09)	0.03	1.22	0.95-1.41	0.69
EMBU					
Father’s rearing style score					
Emotional warmth	0.03(0.06)	0.33	1.03	0.92-1.16	0.57
Punishment	0.01(0.08)	0.03	1.01	0.87-1.18	0.86
Excessive interference	0.24(0.11)	4.79	1.27	1.03-1.57	0.03*
Partialism	−0.23(0.19)	1.48	0.79	0.55-1.15	0.22
Rejection	0.07(0.05)	2.37	1.07	0.98-1.17	0.12
Overprotection	0.25(0.34)	0.53	1.28	0.66-2.49	0.47
Mother’s rearing style score					
Emotional warmth	−0.05(0.06)	0.88	0.95	0.85-1.06	0.35
Punishment	0.89(0.42)	4.38	2.43	1.06-5.58	0.04*
Excessive interference	0.12(0.05)	5.18	1.12	1.02-1.24	0.02*
Partialism	−0.85(0.43)	3.95	0.43	0.19-0.99	0.05
Rejection	−0.00(0.19)	0.00	1.00	0.69-	0.99

SE, standard error; EMBU, egna minnen barndoms uppfostran; PSSS, perceived social support scale; PSS, perceived stress scale; BSMAS, bergen social media addiction scale; VDG-S, video game dependence scale, *p < 0.05.

## Discussion

To our knowledge, in China, this is the first study to analyze the factors influencing the addiction characteristics of NSSI behaviors in hospitalized adolescents with NSSI. Compared to adolescents of the same age who did not exhibit NSSI behaviors, NSSI patients had insufficient emotional warmth from both parents, insufficient social support from within the family, and higher levels of maternal punishment. Previous reports have shown that negative parenting practices are direct or indirect contributors to the development of NSSI behaviors (7, 30). Another cross-sectional study from Brazil found that lack of emotional warmth is significant risk factor for common mental disorders in adolescence (31). Although the findings of these studies are exemplary of our results, significant increases in the prevalence of NSSI among adolescents during the COVID-19 outbreak compared to the pre-epidemic period have been reported in both the United States (32) and China (33). That is, a pure explanation of NSSI behaviors with adolescents’ negative parenting styles from their parents is inadequate. Therefore, to explain this phenomenon in more detail, we conducted a preliminary exploration of the relationship between NSSI and behavioral addiction. The results of our study reported the study group has a higher absolute number of internet addiction and a higher level of internet dependence than the controls. We reported 45.24% of cases with addictive features in the study group and that the behavior was mainly used for internal emotion regulation. In turn, internet dependence is reported to be a known factor associated with NSSI (23). In summary, it is reasonable to assume that both are behavioral addictions, NSSI and Internet addiction may have had a complex interrelationship during the epidemic outbreak, resulting in each being more severe than usual.

Behavioral addiction, the idea that behaviors are self-reinforcing and become repetitive and fixed over time, is affirmed by the four-function model of the NSSI (34). This model assumes that the NSSI is maintained by both positive and negative self-reinforcement processes. These self-reinforcement processes include interpersonal positive reinforcement (producing positive affective or cognitive states and eliciting attention and seeking help), and interpersonal negative reinforcement (reducing negative affective or cognitive states and promoting removal from aversive social situations or reducing interpersonal demands) (35).

We found that female, single-child, video game addiction, excessive interference in father’s rearing style, punishment, and excessive interference in mother’s rearing style are all risk factors for NSSI behavior in adolescents. There is no conclusive information on whether there are gender differences in the prevalence of NSSI in adolescents. A large-scale cross-sectional study from Sweden found that a higher proportion of females reported NSSI behavior (36). But there are no gender differences in the prevalence of NSSI, as reported from non-clinical samples and from studies in the United States (37, 38). Another study showed that NSSI is more common in women aged 16–19 (39). Our research primarily focused on 12–18 years of adolescents with NSSI. In the included cases, it was found that female patients were more likely to have addictive characteristics of NSSI. Wang et al. and Xu et al. previously reported that only children were more likely to have NSSI behavior than adolescents with siblings, because of a lack of social support from society and family (40, 41). Probably the proportion of non-only children was higher in our sample due to the insufficient sample size. However, our analysis shows that the only child is a risk factor for NSSI behavior addiction, confirming the findings of the previous two studies that the only child is more vulnerable to NSSI behavior. Furthermore, we also found that IGD has a predisposing effect on the formation of NSSI behavioral addiction characteristics, confirming that internet “addition” appears to be associated with NSSI (23). More profoundly, we found that internal emotion regulation was higher on the three functional scales of OSIC in the clinical subgroup of NSSI with addiction characteristics. That is, there are reasonable grounds to believe that the addictive characteristics of NSSI function through internal emotion regulation. Excessive interference in the father’s approach, punishment, and undue interference in the mother’s approach all show poor parental styles. Ying et al. reported the relationship between experience of negative parenting practices and NSSI in Chinese adolescents and found a significant positive correlation between negative parenting practices and NSSI (30), which is an important affirmation of our findings. Thus, we conclude that negative parenting styles also negatively contribute to NSSI behavioral addiction traits.

We need additional clarification that NSSI behavior in adolescent is likely to remain an “undifferentiated” form of mental disorder or simply one of the accompanying symptoms of other psychiatric disorders. The available reports also confirm that studies with adolescents with NSSI often correspond to more than a dozen diagnostic names (25, 42). It is the variability and uncertainty of the diagnostic names that has created some obstacles to our study. Therefore, we used monthly and semiannual NSSI frequency as inclusion criteria rather than specific diagnostic names.

There are limitations to this study. The inclusion of too many regression factors in the binary logistic regression model with a relatively small sample size may not be conducive to statistical validity and generalization of the findings. In addition, this study only analyzed the NSSI cross-sectionally and did not give a longitudinal follow-up, making it difficult to draw any causal conclusions.

In conclusion, female, only child, internet addiction, and negative parenting styles were predictors of NSSI behavioral addiction characteristics in adolescents.

## Data availability statement

The raw data supporting the conclusions of this article will be made available by the authors, without undue reservation.

## Ethics statement

The Ethics Committee of the Wuhan Mental Health Center reviewed and approved this study. All participants or their accompanying family members signed informed consent forms to participate.

## Author contributions

JM and XL made substantial contributions to conception and design of the study. JZ drafted the manuscript. RQ and HZ had polished and re-edited the language and logic of the manuscript. YL was responsible for setting up and complement and modify the contents of the manuscript. JM gave final approval of the version to be published. All authors contributed to the article and approved the submitted version.
